# MicroRNA and renal fibrosis in autosomal dominant polycystic kidney disease: a longitudinal study

**DOI:** 10.1007/s40620-024-01965-0

**Published:** 2024-07-05

**Authors:** Silvia Lai, Daniela Mastroluca, Adolfo Marco Perrotta, Maurizio Muscaritoli, Sara Lucciola, Maria Pia Felli, Paolo Izzo, Silverio Rotondi, Sara Izzo, Lida Tartaglione, Roberta Belli, Cesarina Ramaccini, Luciano Izzo, Claudia De Intinis, Valeria Panebianco, Sandro Mazzaferro

**Affiliations:** 1 Nephrology Unit, Department of Translational and Precision Medicine, Sapienza University of Rome, Rome, Italy; 2 Department of Translational and Precision Medicine, Sapienza University of Rome, Rome, Italy; 3 Department of Radiological Sciences, Oncology and Pathology, Sapienza University of Rome, Rome, Italy; 4 Department of Experimental Medicine, Sapienza University of Rome, Rome, Italy; 5 Pietro Valdoni, Department of Surgery, Policlinico Umberto I, Sapienza University of Rome, Rome, Italy; 6 Plastic Surgery Unit, Multidisciplinary Department of Medical-Surgical and Dental Specialties, University of Campania, “Luigi Vanvitelli”, Naples, Italy

**Keywords:** Autosomal dominant polycystic kidney disease, Total fibrotic volume, Plasma aldosterone, Subclinical atherosclerosis parameters

## Abstract

**Background:**

Autosomal dominant polycystic kidney disease (ADPKD) is a hereditary kidney disorder that may progress to kidney failure, accounting for 5–10% of all patients with end-stage kidney disease (ESKD). Clinical data, as well as molecular genetics and advanced imaging techniques have provided surrogate prognostic biomarkers to predict rapid decline in kidney function, nonetheless enhanced tools for assessing prognosis for ADPKD are still needed. The aim of this study was to analyze specific microRNAs involved in the pathogenesis of ADPKD and in the development of renal fibrosis, evaluating their potential role as predictors of renal function loss.

**Methods:**

We evaluated kidney function by estimated glomerular filtration rate (eGFR) in 32 ADPKD patients in different stages of kidney disease at T0 and after a 24-month follow up (T1). Patients were divided into two groups: Rapid disease progression ([RP], n 15) and Non-rapid disease progression ([NRP], n 17), according to the Mayo Clinic classification criteria. At T0, ADPKD patients underwent plasma sampling for quantitative analysis of h-miR-17-5p, h-miR-21-5p and h-miR-199a-5p microRNA expression, using the quantitative reverse transcriptase-polymerase chain reaction (qRT-PCR) method and a 3 T magnetic resonance imaging (MRI), using an advanced MRI imaging protocol, for the quantification of total kidney volume (TKV), total perfusion volume (TPV) and total fibrotic volume (TFV).

**Results:**

The expression of h-miR17-5p was higher (*p* < 0.05) in ADPKD patients with rapid disease progression. h-miR-17-5p, h-miR-21-5p and h-mir-199-5p showed a positive and significant correlation with the eGFR slope (mL/min/1.73 m^2^/year) (*p* < 0.05) but not with the eGFR at both T0 and T1. Both total fibrotic volume (cm^3^) and height-adjusted total fibrotic volume (cm^3^/m) were positively and significantly correlated to h-miR 21-5p and h-miR 199-5p (*p* < 0.05), but not to total kidney volume (cm^3^) and height-adjusted total kidney volume (cm^3^/m).

**Conclusions:**

The microRNAs we studied were associated with fibrosis and renal damage, suggesting their possible role as biomarkers able to identify ADPKD patients at high risk of disease progression regardless of the degree of kidney function, and therefore suitable for medical therapy, and may help uncovering new molecular mechanisms underlying cystogenesis.

**Graphical abstract:**

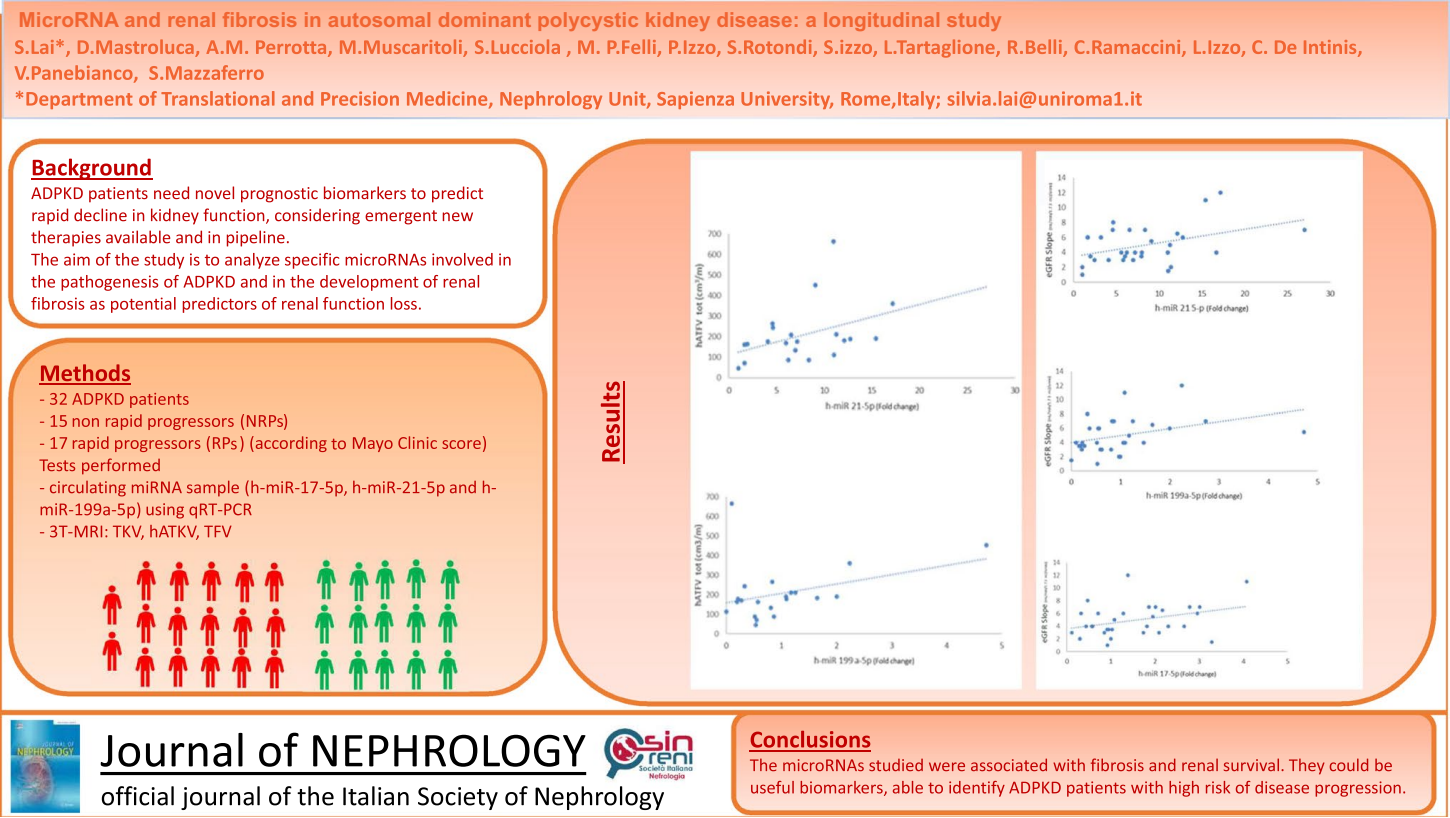

## Introduction

Autosomal dominant polycystic kidney disease (ADPKD) is a genetic kidney disease characterized by multiple and bilateral cystic dilatation of the renal tubules, leading to an increase in total kidney volume (TKV) with progressive disruption of the renal parenchyma and organ failure. It is the most common inherited disorder in the nephrological field, and the genetic prevalence is estimated between 1:400 and 1:1000 live births. ADPKD can arise from mutations in either the PKD1 gene (polycystin 1) or the PKD2 gene (polycystin 2) and, more rarely, in other genes [1]. PKD1-associated disease presents a more severe prognosis and an earlier progression to end-stage kidney disease (ESKD), with a median age at kidney failure of 54 years [2]. Progressive enlargement of the cysts leads to renal parenchyma disruption resulting in an irreversible loss of organ function. However, there is no significant reduction in renal function until total kidney volume is five-fold greater than normal [3]. The primary mechanism of cystic formation is not yet fully known; it is generally accepted that functional loss of polycystins, which enhance hyperproliferative paths mediated by mTOR (mammalian target of rapamycin signaling), cMyc, JAK/STAT (Janus kinases/signal transducer and activator of transcription) and the AMPc/CREB axis (adenosine 3', 5'-cyclic monophosphate/response element-binding protein), is responsible for this process of cystogenesis. Cysts can arise from any tubular portion but originate mainly in the collecting duct. Over time, they detach from the parental tubule and become autonomous, isolated structures filled with fluid deriving from transepithelial secretions. As they expand they tend to lose this connection, probably due to the state of fibrosis of the surrounding interstitium, and the intracystic secretion becomes the only mechanism responsible for the accumulation of fluid in the cysts. The progressive interstitial fibrosis could be the result of cortical ischemia produced by the vascular lesions and luminal narrowing [4, 5]. According to previous studies, tubulointerstitial injury could also be caused by cytokines and chemokines released from inflammatory cells infiltrating the ischemic tissue [1, 4]. Another important pro-inflammatory role could be played by aldosterone. High levels of aldosterone cause the expression of osteopontin, Monocyte chemoattractant protein-1 (MCP-1), Interleukin 6 (IL-6) and IL-1β, and enhance proliferative and fibrogenic pathways, respectively mediated by mitogen-activated protein kinase (MAPK) activity in the nucleus and increasing Transforming growth factor-β (TGFβ) levels [6, 7]. Tubular function impairment and abnormalities in intracellular pathways require novel markers of disease progression, unlike creatinine and glomerular filtration rate (GFR), to define existing kidney damage and predict the progression of disease. In recent years, the emergent role of renal imaging techniques has contributed to identifying novel predictive factors for the evaluation and follow up of renal damage in polycystic kidneys [8]. The use of computed tomography (CT) [9] and ultrasound (US), as well as the evaluation of fibrotic progression using specific Magnetic Resonance Imaging (MRI) sequences is promising considering that gadolinium is not harmful to the kidney [10]. Magnetic resonance imaging is the best method to evaluate kidney volume in this condition, and the relationship between TKV and GFR has been reported in the literature [11]. CRISP (*Consortium for Radiologic Imaging Studies of Polycystic Kidney Disease*) study data demonstrated that TKV corrected for height (haTKV) is more frequently associated with the appearance of complications (arterial hypertension, macrohematuria and proteinuria) and is able to predict the risk of renal function decline, qualifying height-adjusted TKV as a marker of disease progression. Data analysis of 590 ADPKD patients from the Mayo Clinic Translational PKD center who underwent MRI/CT evaluation and three or more estimated GFR (eGFR) evaluations for a follow-up of at least 6 months, led to the creation of a classification that stratified the patients into five different risk classes (A, B, C, D, E) [12]. Using a specific protocol for gadolinium-enhanced MRI makes it possible to estimate the residual parenchymal volume and the total fibrotic volume (TFV) excluding cystic volume. Some studies showed positive correlations between TFV and other markers of cardiovascular disease, as well as kidney disease progression [13, 14]. A search for easy to sample, cheap and non-invasive serum biomarkers needs to be carried out. In recent years, microRNAs (miRNAs) have been associated with epigenetic phenomenon involved in ADPKD etiopathogenesis, able to modulate cyst growth. MicroRNAs are single non-coding RNA molecules, about 22 nucleotides long, capable of regulating gene expression on a post-transcriptional level [15]. They include introns of genes encoding proteins, exons, and introns of non-coding genes. miRNA biogenesis begins at the level of the nucleus where an RNA strand (pri-miRNA) is cut into a miRNA precursor called pre-miRNA. In the cytoplasm, pre-miRNAs are assembled as double stranded RNA molecules which interact with the protein complex Argonaute (Ago 2); this complex is incorporated into ribonucleoproteins called RISC (RNA-induced silencing complex) binding the target mRNA. MiRNAs can have a negative or positive regulator effect, linking elements with partial complementarity mRNA regulators called "seed sequences" located at the 3 UTR, or specific mRNA sequences called MREs (miRNA response elements) [16]. There are limited studies about miRNAs in ADPKD etiopathogenesis. MiRNAs could be implicated both in promoting cyst growth and interstitial fibrosis, both of which are relevant risk factors for chronic kidney disease (CKD) progression. Moreover, some evidence underlines the role of h-miR17-5p, linked to the Wnt/β-catenin-cMyc axis, in cell proliferation and miR-21-5p and miR-199a-5p, known as fibroMir, in pro-fibrosis pathways [17].

The aim of the study was to analyze a set of miRNAs potentially involved in the pathogenesis of ADPKD and in the development of fibrosis, evaluating their potential role as predictors of loss of kidney function and correlating the data to the presence of fibrotic renal tissue.

## Materials and methods

The study protocol was approved by the Local Clinical Research Ethics Committee (CDD25918). The study conforms to the principles outlined in the Declaration of Helsinki and we obtained written consent from each enrolled patient.

### Study design and subjects

We performed a prospective longitudinal study on 32 ADPKD patients consecutively recruited at the University Hospital “Policlinico Umberto I” of Rome, Sapienza University of Rome, Italy. Patients were enrolled between May 2019 and January 2022. Clinical, laboratory and instrumental parameters were evaluated at baseline (T0) and at 24 months (T1).

### Inclusion criteria

Patients aged > 18 years with a diagnosis of ADPKD, defined according to Pei's criteria [18], were eligible. We enrolled both normotensive and hypertensive patients. Arterial hypertension was defined as the use of anti-hypertensive drugs (angiotensin converting enzyme (ACEIs), Angiotensin II receptor blockers (ARBs), beta-blockers, calcium antagonists, alpha1-receptor antagonists and/or diuretics) or by the presence of blood pressure above 140/90 mm Hg in three consecutive measurements.

### Exclusion criteria

We excluded ADPKD patients with: eGFR ≤ 30 ml/min/1.73 m^2^, history of kidney surgery or cyst drainage procedures, other diseases that could potentially affect kidney function, metal clip, pregnancy or nursing, on therapy with tolvaptan, or history of cancer in the previous 5 years. Moreover, patients that refused to give consent or with missing data were also excluded.

### Patients

Patients were divided into two groups: Rapid disease progression (RP, n 15) and Non-rapid disease progression (NRP, n 17), according to the Mayo Clinic classification criteria based on radiologic haTKV classification [12]. Classes 1A and 1B include patients with NRP, while patients with RP are classified as 1C, 1D, 1E to 1E.

### Laboratory measurements

Blood samples were obtained in the morning after 12 h fasting. Fasting plasma glucose (mg/dL), insulin (µU/mL), total serum cholesterol (mg/dL), triglycerides (mg/dL), high-density lipoprotein (HDL, mg/dL), serum creatinine (SCr, mg/dL), serum nitrogen (mg/dL), serum uric acid (SUA, mg/dL), calcium (mg/dL), phosphorus (mg/dL), serum sodium (mEq/L), serum potassium (mEq/L), and C-reactive protein (CRP, μg/L) levels were measured using standard automated techniques. Low-density lipoprotein-cholesterol (LDL, mg/dl) was calculated using the Friedewald equation: TCho − HDL − (Tg/5).

### MicroRNA Analysis

T0 plasma samples were taken for quantitative evaluation of h-miR17-5p, h-miR21-5p and h-miR199a-5p by real-time reverse transcriptase-polymerase chain reaction (RT-PCR) with a single miRNA-specific assay (miRNeasy Serum/Plasma Kit (Qiagen)). H-miR16-5p was used as a normalizer. Starting from aliquots of the frozen plasma, total RNA (including the fraction of microRNA) was extracted using Qiagen miRNeasy serum/plasma kit (Qiagen) according to the manufacturer's instructions (supplementary protocol: Purification of total RNA, including small RNAs, from serum or plasma using the miRNeasy Mini Kit) and with the use of synthetic oligonucleotides cel-miR-39, such as spike-in. Ten ng of RNA was reverse transcribed using the TaqMan ™ MicroRNA Reverse Transcription Kit (Applied Biosystems) according to the manufacturer’s instructions, followed by the pre-amplification reaction (same kit) which allows for uniform amplification of all miRNAs prior to quantification by RT-PCR. For qPCR, pre-amplified 1/5 diluted samples are prepared using TaqMan ADV MIRNA ASSY (miRNA probes) and TaqMan Fast Advance MMIX (both Applied Biosystems) as per the manufacturer's instructions. Relative quantification was performed by the 2-ddCT method normalizing the data with respect to miR16-5p as an endogenous control and using the NT3 (non-fast progressing) sample as a calibrator.

### Magnetic Resonance Imaging (MRI)

All patients underwent an MRI protocol with 3 T magnet (Discovery MR 750, 3T, GE Healthcare) after positioning of the 32-channel surface coil. The acquisition protocol included morphological sequences, single shot T2-weighted (SSFS)(TR 850 ms, TE 105 ms; Flip Angle 90°; FoV 320 × 320; Matrix 320 × 224) acquired on axial, sagittal and coronal planes and sequences Gradient Echo (GRE) T1-weighted (TR 5 ms; TE 1 ms; Flip Angle 15°; FoV 420 × 420; 288 × 192 matrix). To evaluate parenchymal perfusion, we used ultrafast GRE T1-weighted sequences, acquired in the coronal plane (TR 2 mS; TE 1 mS; Flip Angle 13°; Thickness 200 mm; FoV 300 × 300 mm, matrix 192 × 138) during administration of i.v. contrast (Gadobutrol 1 mmol/ml, Gadovist, Bayern) using a perfusion technique, with high temporal resolution of 4 s, for a total duration of about 8 min. The start of dynamic sequences coincides with the administration of i.v. contrast medium [18, 19]. Evaluation of Total Perfusion Volume (TPV) and TFV results from perfusion MRI after a qualitative and quantitative approach. Each parameter was the result of post-processed slice-by-slice renal segmentation respectively in the early arterial phase (1st minute of perfusion) and the late perfusion phase (8th minute of perfusion). Segmentation was guided using colorimetric maps. After segmentation, software Workstation vers. 4.6 was used for 3D volume, resulting in a semiquantitative estimation of vascularized and fibrotic parenchyma. These parameters can estimate the residual functional parenchyma. In the HASTE T2 and T1-weighted 3D GRE morphological images, the following parameters were evaluated: kidney size on 3 planes of acquisition (axial sagittal and coronal), cortical thickness, cortico-medullary differentiation, cyst volume [13]. In the 3D T1 weighted GRE sequences, the morphological characteristics and the signal intensity relating to the presence or absence of high protein content were evaluated, allowing the identification of hemorrhagic cyst content, infected cysts and heteroplastic cysts. MRI evaluation of coronal, axial and sagittal renal diameters allowed us to calculate haTKV and to classify each patient according to class of risk of disease progression according to the Mayo classification [12](http://www.mayo.edu/research/documents/pkd-center-adpkd-classification/doc).

### Statistical analysis

Data management and analysis were performed using IBM® SPSS® Statistics 22.0 for Windows® software (IBM Corporation, New Orchard Road Armonk, New York, United States). The normality of variables was tested using the Shapiro–Wilk method for normal distributions. All continuous variables were expressed as mean ± standard deviation, categorical variables were expressed as number (percentage). Student's t-test was used to determine the between-group difference, and the binomial or Chi-square test was used for the comparison of categorical data. Bivariate correlations and the degree of associations between variables were obtained by Pearson's test. A value of *p* < 0.05 was considered statistically significant.

## Results

**Fig. 1:**
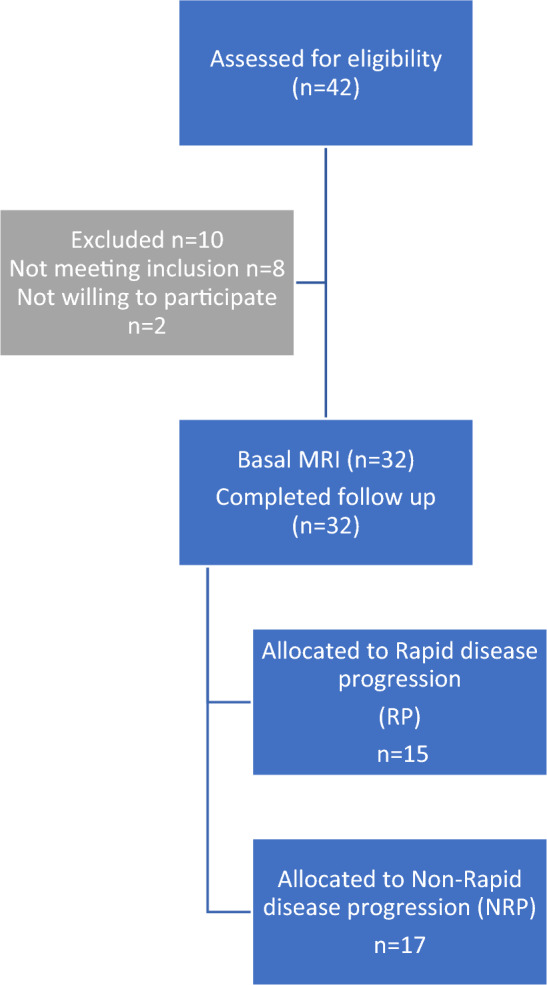
CONSORT diagram; patient enrollment

**Fig. 2:**
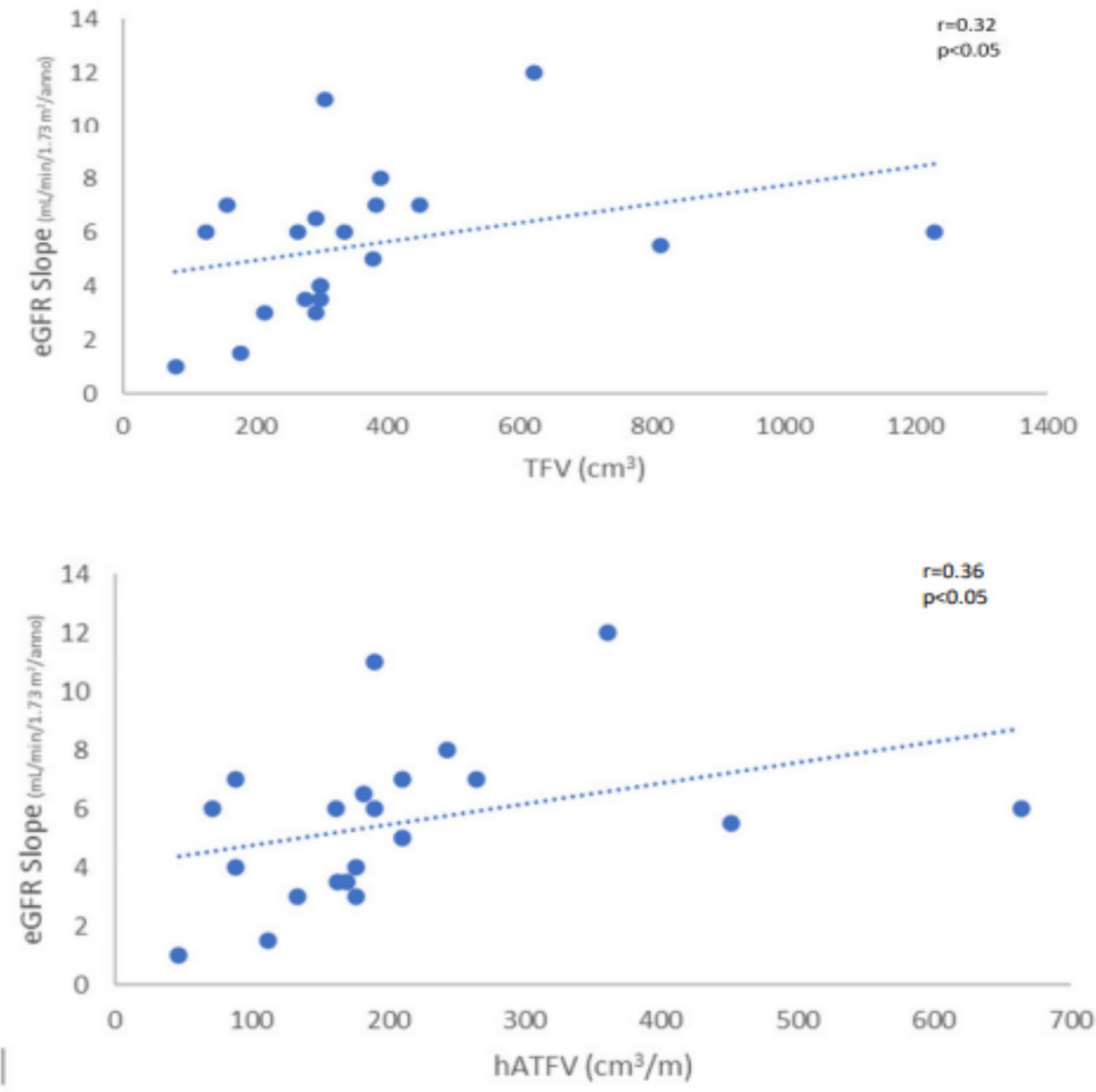
Correlation analysis between total TFV (cm^3^) and haTFV (cm^3^/m) and the eGFR slope (mL/min/1.73 m^2^/year). *eGFR* estimated Glomerular Filtration Rate, *TFV* Total Fibrotic Volume, *haTFV* height-adjusted Total Fibrotic Volume

**Fig. 3:**
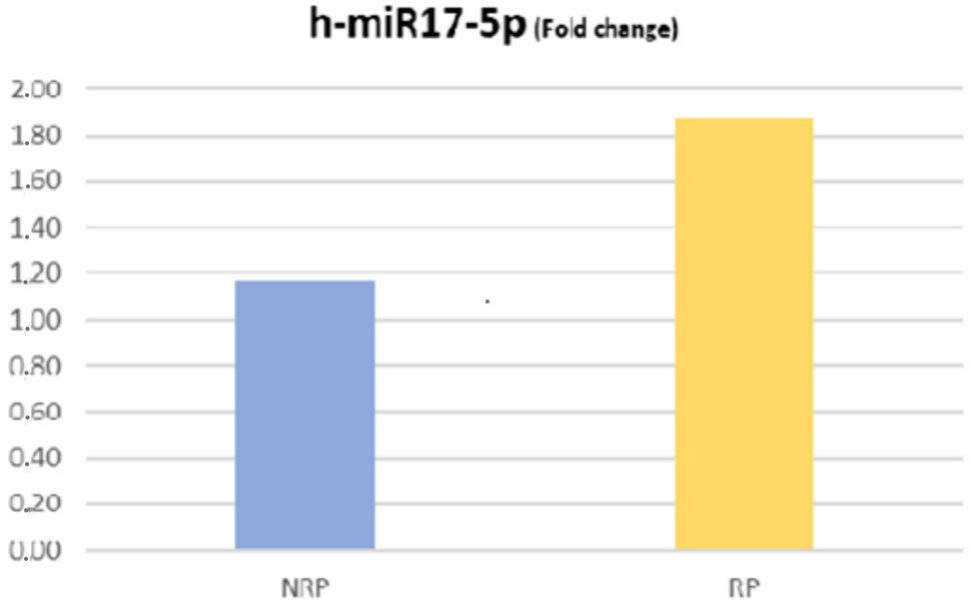
Expression of h-miR-17-5p in ADPKD patients at RP and NRP. H-miR-17-5p was more highly expressed in RP ADPKD patients (RP 1.88 ± 1.04; NRP 1.17 ± 0.87 *p* < 0.05). Results reported in mean ± SD. *ADPKD* autosomal dominant polycystic kidney disease, *RP* rapid disease progression, *NRP* non-rapid disease progression

**Fig. 4:**
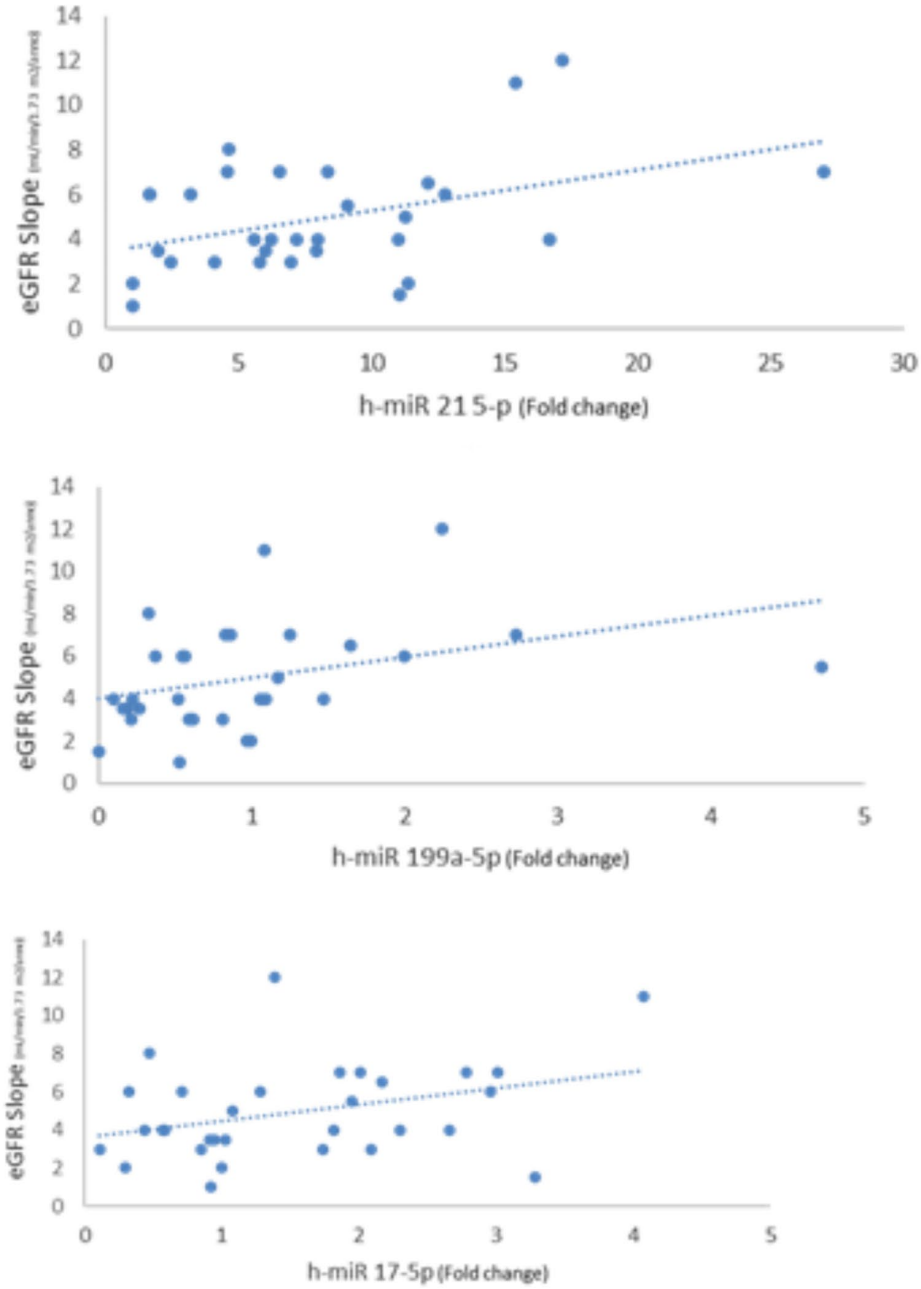
Correlation analysis between h-miR17-5p, h-miR 21-5p and h-miR 199a-5p and the eGFR slope (mL/min/1.73 m^2^/year). H-miR17-5p, h-miR 21-5p and h-miR 199a-5p showed a positive and significant correlation with the eGFR slope (mL/min/1.73 m^2^/year) (respectively, *r* = 0.34, *r* = 0.41, *r* = 0.37, *p* < 0.05). *eGFR* estimated Glomerular Filtration Rate

**Fig. 5:**
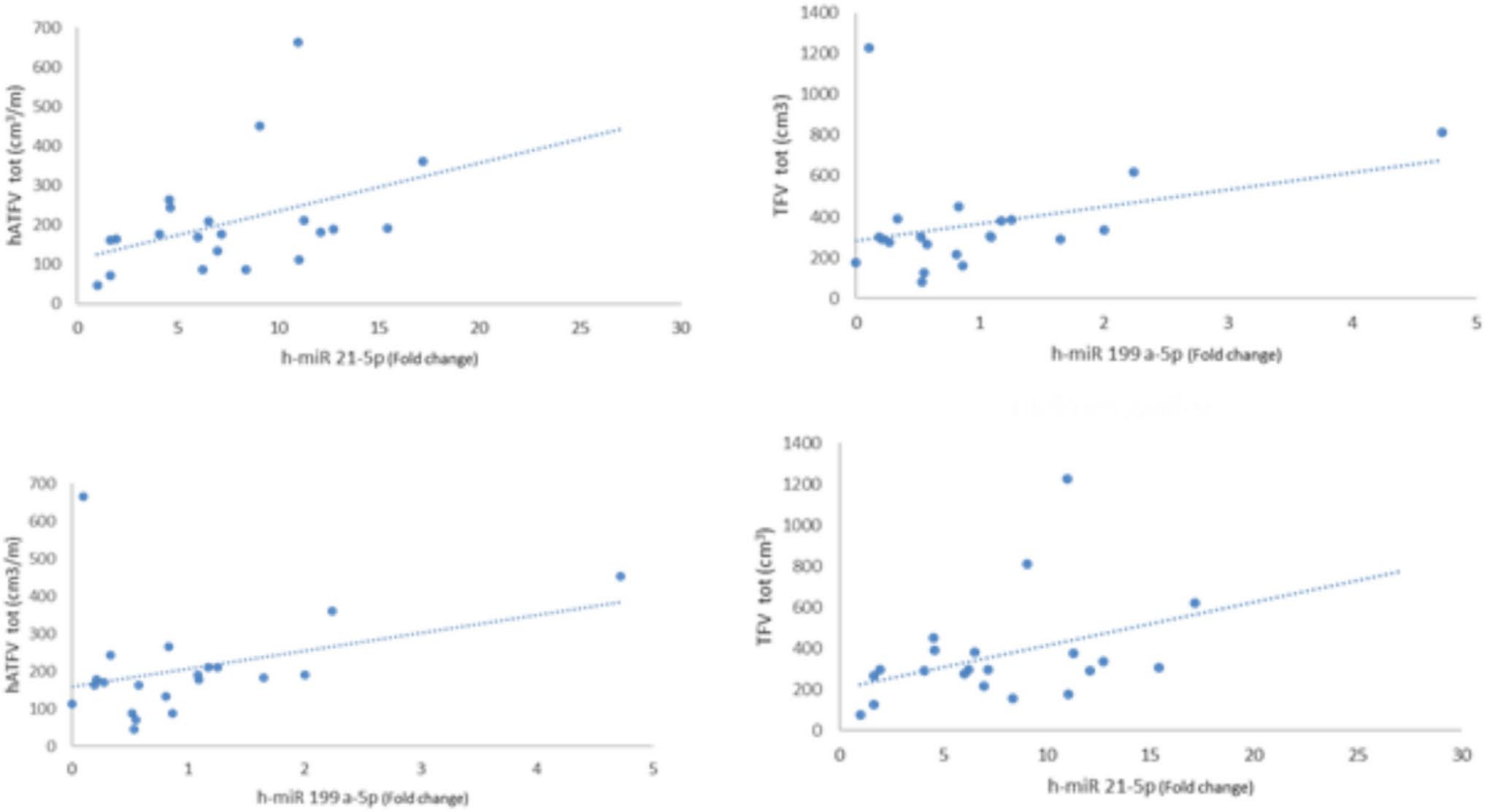
Correlation analysis between, h-miR 21-5p and h-miR 199a-5p and the TFV and haTFV (*r* = 0.38, *p* < 0.05; *r* = 0.40, *p* < 0.0; *r* = 0.34, *p* < 0.05; *r* = 0.36, *p* < 0.05; respectively). H-miR 21-5p and h-miR 199a-5p were positively and significantly correlated with TFV cm^3^ and hATFV cm^3^/m. *TFV* Total Fibrotic Volume, *haTFV* height-adjusted Total Fibrotic Volume

Table 1 shows the individual characteristics of ADPKD patients included in the study. We enrolled 32 ADPKD patients divided into two groups: RP (n:15) and NRP (n;17), calculating haTKV and classifying each patient according to class of risk of disease progression of the Mayo classification (Fig. 1)[15]. Rapid disease progression ADPKD patients showed higher TKV and haTKV values than NRP patients (*p* < 0.001 and *p* < 0.001). The eGFR slope was greater in RP than in NRP patients (*p* < 0.001) as expected. The TFV and the haTFV were not statistically significant in ADPKD patients with RP compared to those with NRP (Table 1). TFV and haTFV showed a significant correlation with the eGFR slope (mL/min/1.73 m^2^/year) (respectively, *r* = 0.32 and *r* = 0.36, *p* < 0.05) (Fig. 2). No correlation was found between TFV and the eGFR at both T0 and T1. The analyzed miRNAs were correlated with each other (*r* = 0.51 for h-miR17-5p and h-miR-21-5p; *r* = 0.43 for h-miR-21-5p and h-miR-199a-5p; *r* = 0.31 for hmiR- 17-5p and h-miR-199a-5p, p < 0.05). Only H-miR-17-5p was more highly expressed in RP patients with respect to NRP patients (Fig. 3, Table 2). No statistically significant difference was found by evaluating the expression of the miRNAs analyzed relating to ADPKD study patients divided by sex (data not shown). H-miR-17-5p, h-miR-21-5p and h-miR-199a-5p showed a positive and significant correlation with the eGFR slope (mL/min/1.73 m^2^/year) (Fig. 4). No correlation was found between the miRNAs analyzed and the eGFR at both T0 and T1. Both h-miR-21-5p and h-miR-199a-5p were found to be positively and significantly correlated to the TFV (*r* = 0.38, *p* < 0.05) and to the haTFV (Fig. 5), but not to total kidney volume and haTFV. The analysis of the correlations between h-miR-17-5p, TFV and haTFV was not found to be statistically significant.

**Table 1:** Characteristics of ADPKD patients included in the study divided into two groups: RP (rapid disease progression) and NRP (non-rapid disease progression)

	RP	NRP	*p* value
Age	43.7 ± 8.51	48.65 ± 10.77	n.s.
Male:Female	10:5	6:11	
Arterial pressure	15 (100%)	17 (100%)	n.s.
ACEi/ARBs	12 (80%)	17 (100%)	n.s.
eGFR CKD-EPI T0 (ml/min/1.73 m ^2^)	55 ± 16	67 ± 25	n.s.
Annual eGFR decline (mL/min/1.73 m ^2^/year)	6.1 ± 2.8	3.87 ± 1.53	< 0.008
TKV tot (cm^3^)	2016.9 ± 761.9	1101.4 ± 416.1	< 0.0002
haTKV (cm^3^/m)	1205.7 ± 404.9	631.7 ± 229.9	< 0.0001
TFV tot (cm^3^)	357.7 ± 175.6	380.5 ± 387.8	n.s.
haTFV (cm^3^/m)	203.4 ± 102.0	215.1 ± 206.7	n.s.
Hb (gr/dl)	12.9 ± 1.42	13.3 ± 1.35	n.s.
Glucose (mg/dl)	86.3 ± 8.70	89.5 ± 15.6	n.s.
Insulin (µU/Ml)	5.42 ± 3.5	5.8 ± 3.5	n.s.
Cholesterol (mg/dL)	200.5 ± 30.7	195.8 ± 51.8	n.s.
Triglycerides (mg/dL)	133.3 ± 57.4	118.6 ± 75.6	n.s.
HDL (mg/dl)	51.4 ± 15.7	56.7 ± 7.9	n.s.
SUA (mg/dl)	6.4 ± 1.2	4.7 ± 0.9	< .007
Calcium (mg/dL)	9.6 ± 0.3	9.4 ± 0.6	n.s.
Phosphorus (mg/dL)	3.4 ± 0.5	3.9 ± 0.4	< .001
Serum sodium (mEq/L)	140.9 ± 2.2	140.9 ± 2.6	n.s.
Serum potassium (mEq/L)	4.5 ± 0.6	4.4 ± 0.4	n.s.
CRP (μg/L)	4.9 ± 6.3	5.0 ± 5.6	n.s.

Results reported as mean ± SD

*eGFR* estimated Glomerular Filtration Rate, *TKV* Total Kidney Volume, *haTKV* height-adjusted Total Kidney Volume, *TFV* Total Fibrotic Volume, *haTFV* height-adjusted Total Fibrotic Volume, *Hb* Hemoglobin, *HDL* high-density lipoprotein, *SUA* serum uric acid, *CRP* C-reactive protein

**Table 2:** Mean quantitative evaluation of h-miR17-5p, h-miR21-5p and h-miR199a-5p in ADPKD patients: RP (rapid disease progression) and NRP (non-rapid disease progression)

	*RP*	*NRP*	*p *value
h-miR-21-5p	7.44 ± 4.02	8.16 ± 6.9	n.s
h-miR-17-5p	1.88 ± 1.04	1.17 ± 0.87	< .05
h-miR-199a-5p	1.11 ± 1.20	0.85 ± 0.66	n.s

Results reported as mean ± SD

RP (rapid disease progression) and NRP (non-rapid disease progression)

## Discussion

Circulating miRNAs may represent the "ideal biomarker" in several diseases since they vary little in the general population and are easy to obtain in a non-invasive way. They can be extracted from cells, tissues and body fluids and are present in human serum [19]. In ADPKD, the abnormal parenchymal architecture of the kidney leads to the activation of the renin– angiotensin–aldosterone system, ischemia, interstitial inflammation and fibrosis. All these events may affect miRNA concentration; in particular h-miR17-5p is linked to the Wnt/β-catenin-cMyc axis and miRNAs, miR-21-5p and miR-199a-5p seem to be involved in pro-fibrotic pathways. Although the aberrant expression of miRNAs exerts a causal pathogenetic role in several diseases, their implication in ADPKD it is still poorly understood [20, 21]. Previous studies have focused on understanding the molecular mechanisms in the pathogenesis of ADPKD, however, there are no data concerning the role of miRNAs in cyst growth and disease progression. Our study is the first to evaluate circulating levels of h-miR-17-5p, h-miR-21-5p and h-miR-199a-5p in patients with ADPKD [22]. ADPKD is a genetic disease characterized by high phenotypic variability regardless of the underlying mutation, thus we need to discover biomarkers able to quantify remaining parenchymal function and provide a prognostic value. Studies conducted on twins affected by ADPKD and on inter- and intra-family variability suggest that other genetic factors affect the severity of the clinical presentation[19–23]. It has been reported that total kidney volume is able to predict the loss of renal function, but the correlation with GFR slope is non-linear except in the later stages of CKD. Our study shows that TKV and haTKV were higher in ADPKD patients defined as RP, however they were not related to eGFR or to the eGFR slope. TFV and haTFV were found to be related to the eGFR slope, an interesting finding considering also the small size of our cohort. MiRNAs h-miR-17-5p, h-miR-21-5p and h-miR-199a-5p were positively related to the eGFR slope. ADPKD patients with RP showed higher values of h-miR-17-5p and greater expression of h-miR17-5p, h-miR- 21-5p and h-miR-199a-5p. Based on the reported results, there are significant correlations between h-miR-21-5p and h-miR-199a-5p, known as "fibro miR", and TFV and haTFV. These data show the potential of the studied miRNAs to identify the risk of disease progression, parenchymal fibrosis and response to treatment. Evidence of the role of these miRNAs can improve knowledge of the pathogenesis of ADPKD and support the development of targeted therapies. Considering the possible role of h-miR-17-5p in the main signaling pathways involved in polycystic disease and the data already described regarding the role of miR-21-5p in other renal diseases such as chronic rejection or diabetic nephropathy, both seem to be key players in cell proliferation and renal fibrosis, regardless of the underlying disease. [22–24]. The use of antagonists of these miRNAs could be a future therapeutic option. RGLS4326 has already proven to be a safe, stable drug for the treatment of ADPKD, and effective in slowing the growth of cysts in PKD mouse models. A clinical trial is currently ongoing to evaluate the use of antiMIR17 in 19 molecules in ADPKD patients (ClinicalTrials.gov identifier NCT04536688).

The main limitation of this study is that it was conducted in a small cohort of ADPKD patients. Moreover, the protocols used to analyze miRNAs and quantify fibrotic volume are presently limited to specialized centers with advanced laboratory and imaging techniques. Further studies on larger populations are necessary to confirm our results ultimately leading to new therapeutic pathways.

## Conclusion

The miRNAs we analyzed were associated with fibrosis and renal survival, suggesting their possible role as biomarkers able to identify ADPKD patients at high risk of disease progression, regardless of their renal function. The use of miRNAs could further represent a target of therapy response, and help further defining molecular mechanisms underlying cystogenesis.

## Data Availability

All data generated or analyzed during this study are included in this published article (and its supplementary information files).
